# PACIFIC‐5 Trial: Refining Patient Selection for Consolidation Durvalumab in Unresectable Stage III NSCLC

**DOI:** 10.1111/1759-7714.70305

**Published:** 2026-07-26

**Authors:** Shiqiong Zhou, Qinghua Ke

**Affiliations:** ^1^ Department of Chemoradiotherapy Jingzhou First People's Hospital Jingzhou Hubei Province China

To the Editor,

1

We read with great interest the phase III PACIFIC‐5 trial by Wu et al., which reported a significant progression‐free survival (PFS) benefit with consolidation durvalumab versus placebo in patients with unresectable stage III NSCLC without progression after either concurrent or sequential chemoradiotherapy (cCRT or sCRT) [1]. The study elegantly extends the PACIFIC paradigm to a broader, more real‐world population, including those receiving sCRT. Nevertheless, several clinically relevant issues regarding patient selection and biomarker interpretation deserve further discussion to optimize the application of this therapy.

The lack of a clear PFS benefit in key subgroups—particularly patients with tumor PD‐L1 expression < 1% (hazard ratio [HR] = 0.97, 95% confidence interval [CI]: 0.65–1.45) and those with non‐squamous histology (HR = 1.07, 95% CI: 0.80–1.43)—generates hypothesis‐forming observations. The point estimate for PD‐L1 < 1% is essentially neutral, while the HR of 1.07 for non‐squamous tumors actually favors placebo. In the original PACIFIC trial, durvalumab also showed limited benefit in the PD‐L1 < 1% subgroup (HR for PFS around 0.73 but with wide CIs crossing 1.0), and overall survival benefits were less pronounced in squamous histology. Thus, the PACIFIC‐5 findings align with the original trial rather than contradict it, suggesting that these subgroups may derive less absolute benefit from consolidation immunotherapy. We acknowledge that PACIFIC‐5 was not statistically powered for subgroup analyses, and the confidence intervals for these estimates crossed 1.0. The authors interpreted these null findings cautiously, attributing them to imbalances in baseline characteristics and small sample sizes. Still, the biological basis for the seemingly poor efficacy in non‐squamous tumors remains unclear; potential explanations include differences in genetic mutation profiles (e.g., higher frequency of EGFR, ALK, or KRAS mutations in non‐squamous histology) that may affect the immune microenvironment and response to PD‐L1 blockade. These observations warrant further mechanistic exploration.

While the PFS benefit in the sCRT subgroup (HR = 0.75, 95% CI: 0.58–0.97) is encouraging, the notable difference in subsequent anticancer therapy between arms (54.3% in the placebo arm vs. 37.7% in the durvalumab arm) is an expected consequence of subsequent standard‐of‐care immunotherapy after progression—no protocol‐mandated crossover was allowed. Specifically, 17.8% of placebo patients received subsequent immunotherapy compared to 9.5% in the durvalumab arm. Moreover, subsequent chemotherapy was also more common in the placebo arm (38.8% vs. 31.3%). The higher rate of subsequent therapy in the placebo arm is not due to protocol bias but rather reflects real‐world practice: patients who progress on placebo are eligible for approved immunotherapies (e.g., durvalumab or anti‐PD‐1 agents), whereas those who progress on durvalumab have fewer proven options. This imbalance inevitably dilutes the observed overall survival (OS) benefit, as reflected in the non‐significant OS HR of 0.87 (95% CI: 0.66–1.15, *p* = 0.346). We commend the authors for transparent reporting. The planned final OS analysis with longer follow‐up will provide a more reliable estimate of durvalumab's true OS benefit, particularly in the sCRT population where follow‐up is ongoing.

The suggestion to use circulating tumor DNA (ctDNA) clearance as a complementary biomarker is promising but faces practical hurdles. Current limitations include high costs, lack of assay standardization across platforms, variable turnaround times, and limited accessibility in community settings. Moreover, ctDNA negativity does not always equate to molecular remission, and false positives can occur. Thus, while ctDNA kinetics may help refine patient selection within PD‐L1‐negative or non‐squamous subgroups, prospective validation and harmonization of assays are needed before clinical implementation [2].

In summary, PACIFIC‐5 represents a landmark advance. Given the trial's limited power to assess efficacy in PD‐L1‐negative and non‐squamous subgroups, future hypothesis‐generating studies should explore dynamic biomarkers such as ctDNA clearance during chemoradiotherapy, noting that ctDNA kinetics may differ between histological subtypes. Non‐squamous tumors often exhibit higher tumor mutational burden and distinct shedding dynamics compared with squamous tumors, potentially influencing molecular response assessment. Similarly, PD‐L1‐negative patients with early ctDNA clearance might still derive benefit from consolidation immunotherapy, suggesting that ctDNA could serve as a complementary selection tool within PD‐L1‐negative patients rather than a substitute for PD‐L1 testing [2]. Addressing these questions will further solidify the role of consolidation immunotherapy in this heterogeneous disease.

## Author Contributions

**Qinghua Ke:** conceptualization, formal analysis, writing – original draft, writing – review and editing. **Shiqiong Zhou:** conceptualization, formal analysis, writing – review and editing, writing – original draft.

## Funding

The authors have nothing to report.

## Conflicts of Interest

The authors declare no conflicts of interest.

## Data Availability

Data sharing not applicable to this article as no datasets were generated or analyzed during the current study.
